# Lifelong Learning in the Educational Setting: A Systematic Literature Review

**DOI:** 10.1007/s40299-023-00738-w

**Published:** 2023-05-13

**Authors:** Win Phyu Thwe, Anikó Kálmán

**Affiliations:** 1 Doctoral School of Education, University of Szeged, Szeged, Hungary; 2 Department of Technical Education, Faculty of Economic and Social Sciences, Budapest University of Technology and Economics (BME), Műegyetem rkp.3, H-1111, Budapest, Hungary

**Keywords:** Lifelong learning, Lifelong learning skills, Lifelong learning competencies, Systematic literature review

## Abstract

This systematic literature review aimed to provide updated information on lifelong learning in educational research by examining theoretical documents and empirical papers from 2000 to 2022. This review sought to identify concepts, theories, and research trends and methods linked to lifelong learning in educational research in different countries. Our review findings showed that theoretical papers, such as reports, policies, and concepts of lifelong learning, are generally much more extensive than empirical studies. Word cloud analysis revealed that the most prominent concepts were lifelong learning skills, lifelong learning competencies, and the three types of lifelong learning (formal, nonformal, and informal). Following the inductive analysis, this study investigated three common research trends: conceptual framework or policies of lifelong learning, lifelong learning abilities, and influencing factors of lifelong learning and/or lifelong learning abilities. Regarding methodology, this study identified only three studies that used mixed methods, which is insufficient in the field. In addition, heterogeneity was also observed between research instruments in lifelong learning. Different data analysis techniques can be applied in this field, including content analysis, descriptive analysis, and inferential analysis. Finally, the participants involved in the examined studies were students, primary and secondary school teachers, undergraduates, postgraduates, student teachers, European Union Lifelong Learning experts, young adults, teacher educators, administrators, and academic staff.

## Introduction

Lifelong learning is a broad term whose definitions have common meanings and which has been explained by organizations such as the European Commission, the United Nations Educational, Scientific and Cultural Organization (UNESCO), and the Organization for Economic Cooperation and Development (OECD).

The European Commission (2001) defines lifelong learning as any intentional learning activities conducted throughout a person’s lifetime to improve their knowledge, skills, and competencies from an individual, municipal, societal, and/or career standpoint. From this conventional definition, a more robust definition of lifelong learning emerged—that is, lifelong learning refers to all processes that transform a person’s body, mind, and social experiences intellectually, emotionally, and practically before they are integrated into their life story, resulting in a more experienced individual (Jarvis, 2009).

Meanwhile, the UNESCO definition of lifelong learning includes all intentional learning from birth to death that attempts to advance knowledge and skills for anyone who intends to engage in learning activities. Part of the broad definition of lifelong learning refers to both informal learning in settings such as the workplace, at home, or in the community and formal education in institutions such as schools, universities, and alternative education centers (Tuijnman et al., 1996). According to the European Lifelong Learning Initiative, lifelong learning is a consistently supportive process that stimulates and empowers individuals in acquiring all the awareness, values, skills, and comprehension they would require throughout their lifetime and apply them with self-belief, innovation, and pleasure in all positions, contexts, and climates (Watson, 2003). Therefore, lifelong learning can be generally defined as learning that one seeks throughout their life and that is flexible, varied, and accessible at diverse times and locations.

According to John Dewey, education is the process of giving a person the skills necessary to take charge of their world and fulfill their obligations. The ideas of education and lifelong learning endure over the life of an individual's existence. Lifelong learning transcends the limits of education and goes beyond traditional education (Edwards & Usher, 1998). In this regard, it is vital to assess how education settings can support lifelong learning. This literature review is the groundwork for the future implementation of educational institutions as lifelong learning centers.

## Importance of a Systematic Literature Review of Lifelong Learning

A review of educational research in lifelong learning is the initial step to understanding relevant concepts and conducting empirical research. Both narrative and systematic reviews help identify research gaps and develop research questions, respectively. Meanwhile, systematic reviews include not only information obtained from the literature but also the adopted approach and where and how the literature was found. The significance of a systematic literature review (Cronin, 2011; Mallett et al., 2012) can be seen in the criteria used to assess whether to include or exclude a study from the review, reducing article selection bias.

Do et al. (2021) conducted the first systematic scientific investigation of the literature on lifelong learning although the selected studies focused only on the Southeast Asia context. Because the researchers used bibliometric analysis, it was not possible to study the intricacies of a lifelong learning issue, evaluate the quality of each scientific paper, or accurately highlight its effects on the topic. To overcome these limitations and provide a more general overview of the research topic, another systematic review of lifelong learning literature must be conducted. Therefore, our research will contain policy document, theoretical and empirical papers from 2000 to 2022 to provide updated information on lifelong learning in educational research. This literature review aims to identify concepts and theories, research areas, research trends, and research methods associated with lifelong learning in educational research in different countries. These intentions have guided the following research questions for this literature review:

1. What concepts and theories have been applied to explain lifelong learning in education research?
2. What research problems have been examined in lifelong learning in education research?
3. What research methodologies have been adopted to evaluate lifelong learning in education?

## Methodology

Lifelong learning in the educational setting is assessed using a systematic review of literature instead of a narrative review or bibliometric analysis. A systematic literature review is considered as a scientific, unambiguous, and repeatable process for locating, analyzing, and summarizing every available published and registered research article to address a clearly articulated question (Dewey & Drahota, 2016). To ensure the effectiveness of the document search strategy, this study used the Preferred Reporting Items for Systematic Reviews and Meta-Analyses (PRISMA 2020) as suggested by Page et al. (2021).

### Procedure

This study employed the largest multidisciplinary databases, such as Web of Science (WoS), Scopus, and ProQuest, to search for studies in lifelong learning. It also investigated two institution-based websites focusing on lifelong learning, the UNESCO Institute of Lifelong Learning and the European Commission, and gathered their policy documents, publications, and reports. Throughout the period 2000–2022, all lifelong learning studies were considered to ensure that all up-to-date information is captured. Our keywords were “lifelong learning” and “education,” and we set our filters to include open-access articles and journals related to education, social science, and the English language. Based on the publication of hundreds of articles, we developed our inclusion and exclusion criteria.

### Included and Excluded Studies

We selected articles based on the following criteria: published in educational science and social science publications, employed both theoretical and empirical research (qualitative, quantitative, or mixed methods), and open access. The decision was made to exclude lifelong learning articles that did not focus on the education field, such as medicine, engineering, and labor studies, and those with unsuitable titles and abstracts. Duplicate articles were removed after the articles that met these criteria were assessed using R Studio software.

### Screening

**Fig. 1:**
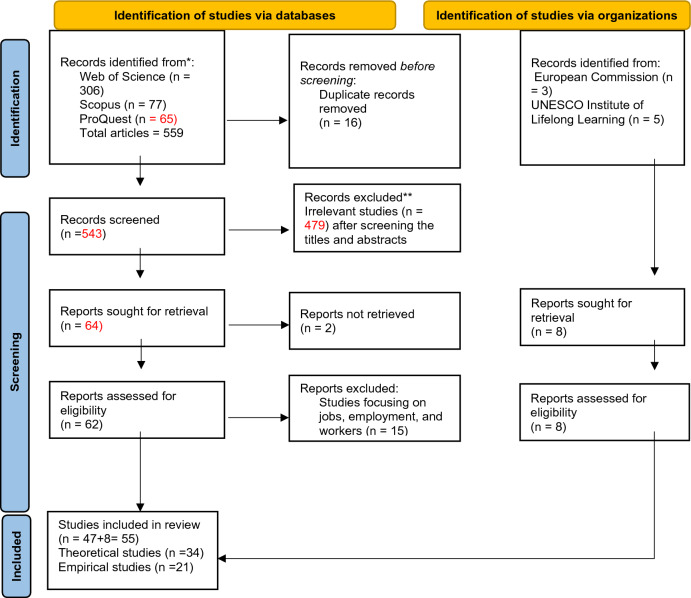
Selection procedure of studies for analysis according to PRISMA 2020

The screening stage involved an evaluation of titles and abstracts to determine their suitability for the research question and literature review methodology. Through this method, we discovered irrelevant articles and removed them. The remaining policy documents, theoretical and empirical studies were reviewed and analyzed in the last screening round, producing a total of 55 eligible articles. Figure 1 shows the procedure of finding and selecting relevant literature according to the PRISMA 2020 flow diagram (Page et al., 2021).

### Data Extraction and Analysis

To answer the research questions, we categorized lifelong learning concepts and theories, research trends, and methods. We extracted the concepts and theories from both policy documents, theoretical and empirical publications and then gathered information on research trends and methods based on empirical studies. We then conceptually coded and categorized the data and used R Studio software to analyze the articles both qualitatively and quantitatively.

## Findings

### Lifelong Learning Concepts and Theories

**Fig. 2:**
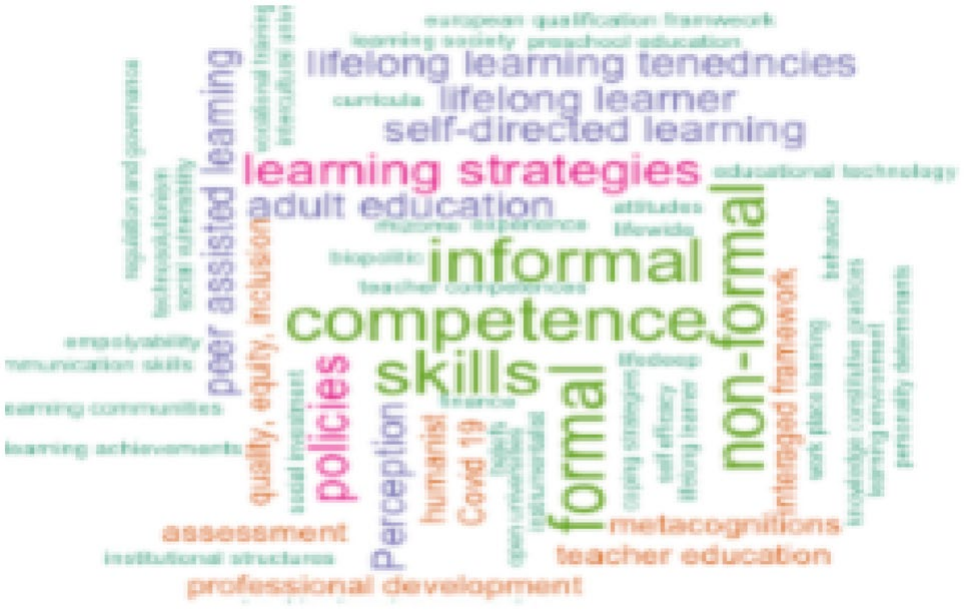
Word cloud analysis of lifelong learning concepts

Our analysis of 55 studies covering the period 2000–2022 showed that lifelong learning was explained using different concepts based on the research area and trends. An overview of concepts related to lifelong learning can be found in Table 1. Meanwhile, the results of the word cloud analysis in R Studio (Fig. 2) revealed that the most prominent concepts were lifelong learning skills, lifelong learning competencies, and the three types of lifelong learning (formal, nonformal, and informal).

**Table 1:** Analysis of concepts related with lifelong learning

Concepts	Authors
Adult education	Ivanova (2009); Mandal (2019); Tsatsaroni & Evans (2014)
Assessment	Green (2002); Matsumoto-Royo et al. (2022)
Attitudes toward learning and individual lifelong learning behavior	Lavrijsen & Nicaise (2017)
Beliefs	Bath & Smith (2009)
biopolitical shift of lifelong learning	Beighton (2021)
Communication skills and predisposition	Deveci (2019)
Coping strategies	Muller & Beiten (2013)
COVID-19	Deveci (2019); Eschenbacher & Fleming (2020)
Educational technology	Sen & Durak (2022)
European qualification framework	Elken (2015)
Finance	Oosterbeek & Patrinos (2009)
Humanism	Black (2021); Osborne & Borkowska (2017)
Integrated framework of lifelong learning	James (2020); Panitsides (2014)
Intercultural universities	Tyson & Vega (2019)
Knowledge-constitutive practices	Nicoll & Fejes (2011)
Learning achievements	Omirbayev et al. (2021)
Learning strategies	Cort (2009); Muller & Beiten (2013)
Life-deep learning, ethical principles, learning society, and learning communities	Osborne & Borkowska (2017)
Lifelong learners	Adams (2007); Bagnall (2017); Bath & Smith (2009)
Lifelong learning competencies	Council of the European Union (2018); Grokholskyi et al. (2020); Kwan et al. (2017); Omirbayev et al. (2021); Shin & Jun (2019)
Lifelong learning experience	Shin & Jun (2019)
Lifelong learning policies	Rambla et al. (2020); Tuparevska et al. (2020a, 2020b); Valiente et al. (2020a, 2020b)
Lifelong learning skills	Adams (2007); Bath & Smith (2009); Deveci (2022); Karataş et al. (2021); Moore & Shaffer (2017); Omirbayev et al. (2021)
Lifelong learning tendencies	(Matsumoto-Royo et al. (2022); Nacaroglu et al. (2021); Sen & Durak (2022)
Metacognitions	Grokholskyi et al. (2020); Matsumoto-Royo et al. (2022)
Open universities	Zuhairi et al. (2020)
Peer-assisted learning	Kuit & Fildes (2014)
Perception	Adams (2007); Buza et al. (2010); Cefalo & Kazepov (2018)
Personal learning environment	Yen et al. (2019)
Personality determinants	Grokholskyi et al. (2020)
Preschool education	Karalis (2009)
Professional development	Theodosopoulou (2010); Zuhairi et al. (2020)
Quality, equity, and inclusion	Sunthonkanokpong & Murphy (2019)
Regulation and governance, institutional structures, and curricula	Green (2002)
Rhizome	Usher (2015)
Self-directed learning	Karataş et al. (2021); Kuit & Fildes (2014); Nacaroglu et al. (2021)
Self-efficacy	Sen & Durak (2022)
Social exclusion	Tuparevska et al. (2020a, 2020b)
Teacher competencies	Theodosopoulou (2010)
Teacher education	Simmons & Walker (2013); Sunthonkanokpong & Murphy (2019)
Teaching–learning approaches	Karataş et al. (2021)
Techno-solutionism and instrumentalism	Black (2021)
Three types of lifelong learning (formal, nonformal, and informal)	do Nascimento et al. (2018); UIL (2017); Walters et al. (2014); Yang et al. (2015); Yen et al. (2019); Yorozu (2017)
Workplace learning	Maxwell (2014)

Many publications included in our review lack a clear theory of lifelong learning. Our analysis of the 55 studies, however, revealed an attempt by scholars to apply comprehensive theory (Bagnall, 2017), theory of transformative learning (Eschenbacher & Fleming, 2020), theories of societal learning (Osborne & Borkowska, 2017) to lifelong learning.

### Research Areas in Lifelong Learning

We inductively analyzed 21 of the 55 empirical studies in our review to examine the common research problems that the researchers presented and addressed. From this analysis, three common research areas emerged: problems associated with the conceptual framework or policies of lifelong learning, issues surrounding lifelong learning abilities, and challenges linked to factors that influence lifelong learning and/or lifelong learning abilities. Table 2 presents a detailed analysis of these research problems in the 21 studies.

**Table 2:** Analysis of research areas

Research areas		Authors
Concepts used in policies of lifelong learning	A conceptual framework for lifelong learners who leave school	Adams (2007)
	The notion of social isolation in lifelong learning policies developed by the European Union (EU)	Tuparevska et al. (2020a)
	Vulnerability in lifelong learning policies developed by the EU	Tuparevska et al. (2020b)
	The relations between lifelong learning policies and the definition of young adults in terms of social vulnerability	Rambla et al. (2020)
Lifelong learning abilities	Lifelong learning skills during the course	Moore & Shaffer (2017)
	Lifelong learning skills in biology	Kuit & Fildes (2014)
	Lifelong learning tendencies, technical self-efficacy, and professional competence	Sen & Durak (2022)
	The mediating function of preparedness for self-directed learning in the correlation between lifelong learning skills and preservice teachers’ teaching–learning style	Karataş et al. (2021)
	Different forms of teacher training in lifelong learning	Simmons & Walker (2013)
	Relation between lifelong learning tendencies and self-regulatory skills	Nacaroglu et al. (2021)
Factors that influence lifelong learning and/or lifelong learning abilities	Multi-layered influence of individual and organizational factors on lifelong learning competencies	Shin & Jun (2019)
	Characteristics and traits that may indicate a person’s tendency for lifelong learning	Bath & Smith (2009)
	Importance of external barriers to explain inequalities in lifelong learning participation	Lavrijsen & Nicaise (2017)
	Personal learning environment (PLE) management’s forecast of PLE application in fostering lifelong learning	Yen et al. (2019)
	Interpersonal communication in the learning and teaching environment as a key indicator of current and future engagement in lifelong learning	Deveci (2019)
	Role of personality traits and metacognitions in the acquisition of lifelong learning competency	Grokholskyi et al. (2020)
	Impact of the pandemic on lifelong learning skills	Deveci (2022)
	Assessment processes that foster the improvement of metacognition abilities and encourage lifelong learning	Matsumoto-Royo et al. (2022)
	Challenges to improve lifelong learning in open universities	Zuhairi et al. (2020)
	Learning strategies of lifelong learners	Muller & Beiten (2013)
	How education can be organized to ensure quality and lifelong learning	Buza et al. (2010)

We also found that researchers described lifelong learning abilities using terms such as “lifelong learning skills,” “lifelong learning competencies,” and “lifelong learning tendencies.” Some studies also investigated the impacts of demographic data to address their research problems (e.g., Buza et al., 2010; Nacaroglu et al., 2021; Sen & Durak, 2022; Shin & Jun, 2019).

### Research Methodologies in Lifelong Learning

Of the 21 studies, 11 conducted quantitative research, seven qualitative researches, and three mixed-method research. Differences were observed in their research instruments, analysis, and participants based on their research design and methods. We will discuss these research methodologies based on the aforementioned three common research problems.

Table 3 summarizes the main research instruments used by lifelong learning studies. The researchers also adopted several other research tools, including the Competences Scale for Educational Technology Standards, the Teaching–Learning Conceptions Scale, the Self-Directed Learning Readiness Scale, the Perceived Self-Regulation Scale, the Dimension Learning Organization Questionnaire, learning agility, knowledge sharing, learning approaches, the General Self-Efficacy Scale, the Openness to Experience Scale, change readiness, the Epistemic Beliefs Inventory, general intelligence, self-assessment of metacognitive knowledge and metacognitive activity, reflexive skills, the questionnaire of implicit theories, a diagnosis of motivational structure, and the teaching and assessment strategies for pedagogical practice instrument, to investigate the relation between lifelong learning abilities and other variables or their impacts.

**Table 3:** Analysis of research instruments based on their research problems

Research problems	Research instruments	Authors
Conceptual framework or policies of lifelong learning	Interview	Adams (2007)
	Interviews, documents	Tuparevska et al. (2020a)
	Interviews, policy documents	Tuparevska et al. (2020b)
	Interview	Rambla et al. (2020)
Lifelong learning abilities	Effective Lifelong Learning Inventory	Moore & Shaffer (2017)
	Student surveys	Kuit & Fildes 2014)
	Lifelong learning tendencies scales	(Sen & Durak (2022)
	Lifelong Learning Tendency Scale	Karataş et al. (2021)
	Interview	Simmons & Walker (2013)
	Lifelong learning scale, semi-structured interviews	Nacaroglu et al. (2021)
	Lifelong learning competencies scales	Shin & Jun (2019)
Factors that influence lifelong learning and/or lifelong learning abilities	Lifelong learning scale	Bath & Smith (2009)
	Data from the Program for the International Assessment of Adult Competencies	Lavrijsen & Nicaise (2017)
	Personal environment learning	Yen et al. (2019)
	Predispositions for Lifelong Learning	Deveci (2019)
	Questionnaire form on the individual experience of LLL, development of LLL competency (scores of two semesters)	Grokholskyi et al. (2020)
	Lifelong learning skills	Deveci (2022)
	“Metacognition and Lifelong Learning in the Teaching and Assessment of Future Teachers” questionnaire, interview	Matsumoto-Royo et al. (2022)
	Interviews, focus group discussion	Zuhairi et al. (2020)
	Learning styles instrument, coping strategies scale	Muller & Beiten (2013)
	Lifelong learning conception, the relation between teaching and lifelong learning, interview	Buza et al. (2010)

In some cases, some researchers developed these instruments, while in others, they modified existing tools (e.g., Effective Lifelong Learning Inventory (Crick et al., 2004), Lifelong Learning Competencies Scale (Sahin et al., 2010), and Lifelong Learning Tendency Scale (Coşkuna & Demirel, 2010)). These researchers also performed many types of data analysis based on their data collection tools and data distribution methods, including descriptive and diagnostic analyses, hierarchical linear modeling, reliability, principal component analysis, confirmatory factor analysis, structural equation modeling, regression, multivariate regression, correlation, comparative analyses (*t*-test or Mann–Whitney *U* test), and content analysis.

These studies also involved several types of participants, such as students, primary and secondary school teachers, undergraduates, postgraduates, student teachers, EU Lifelong Learning experts, young adults, teacher educators, administrators, and academic staffs, which all represent different contexts. Table 4 shows that Asia, the Middle East, and Europe can be regarded as the general contexts of these studies. Notably, however, fewer studies have been conducted in Asia than in the Middle East and Europe, which may pose a challenge to the generalization of the findings of some studies in these contexts.

**Table 4:** Analysis of participants based on research problems

Research problems	Participants	Context	Authors
Conceptual framework or policies of lifelong learning	Secondary school teachers	Australia	Adams (2007)
	EU LL experts	Europe	Tuparevska et al. (2020a)
	EU LL experts	Europe	Tuparevska et al. (2020b)
	Young adults, experts	Spain, Austria	Rambla et al. (2020)
Lifelong learning abilities	Undergraduate students	United States	Moore & Shaffer (2017)
	Undergraduate students	Australia	Kuit & Fildes (2014)
	Student teachers	Turkey	Sen & Durak (2022)
	Student teachers	Turkey	Karataş et al. (2021)
	Teacher educators	England	Simmons & Walker (2013)
	Students	Turkey	(Nacaroglu et al. 2021)
Factors that influence lifelong learning and/or lifelong learning abilities	Primary teachers	Korea	Shin & Jun (2019)
	University students	Australia	Bath & Smith (2009)
	Students	Europe	Lavrijsen & Nicaise (2017)
	Master students	United States	Yen et al. (2019)
	Students and teachers	United Arab Emirates	Deveci (2019)
	Students	Ukraine	Grokholskyi et al. (2020)
	Students	United Arab Emirates	Deveci (2022)
	Student teachers	United States	Matsumoto-Royo et al. (2022)
	Administrators, academic staff, students	Taiwan, Indonesia	Zuhairi et al. (2020)
	Students	Denmark, Finland, Germany	Muller & Beiten (2013)
	Postgraduate students and teachers in teacher education	Albania	Buza et al. (2010)

## Discussion

The results of our review showed that theoretical papers, such as reports, policy document, and lifelong learning concepts were generally much more extensive than empirical studies. Despite attempts to formulate new lifelong learning theories and apply existing ones, researchers have yet to develop a strong theory of lifelong learning. Consistent with the results of our systematic review is Steffens (2015) assertion that no single theory of learning can adequately account for all types of lifelong learning.

The prior studies' use of lifelong learning concepts can be the basis for further studies to build comprehensive theoretical frameworks in line with the current situation. This study’s concept analysis identified lifelong learning skills; lifelong learning competencies; and formal, nonformal, and informal learning as the most salient concepts.

Meanwhile, the analysis of each empirical study’s research problems generated three shared research trends in lifelong learning. Additionally, these studies were found to have investigated the relation between lifelong learning abilities and other variables, such as professional competencies, self-efficacy, and teaching–learning approaches. Moreover, they examined the factors affecting lifelong learning, lifelong learning skills, lifelong learning competencies, and lifelong learning tendencies; the hierarchical effects of individual and organizational variables; external barriers; professional learning environment; metacognitions; and personality determinants. Alongside these factors, demographic components such as gender, age, subjects, and educational level can also significantly influence lifelong learning. Furthermore, this review also found research gaps in lifelong learning in educational research, which offers the potential to explore lifelong learning using variables such as new learning communities, advanced teaching–learning techniques, learning styles, learning strategies and motivation in addition to self-directed learning, personal learning environments, and educational technology.

With regard to research methods, this study identified only three studies that used mixed methods, indicating an inadequacy in the field. Hence, all future research of lifelong learning should be conducted using mixed methods. Our examination of instruments revealed different tools that were used to assess the three common research problems. Such an effort may require the application of different data analysis techniques, including content analysis, descriptive analysis, and *inferential analysis.*

The prior studies, as a result of our review, only interviewed lifelong learning specialists, young adults, and secondary teachers to address their research issues, such as concepts and policies. Indeed, the development of lifelong learning policies or conceptual frameworks would benefit from the involvement of teachers from basic education schools, teacher education institutions, and universities.

Several research problems associated with lifelong learning capabilities involved university students, students and teacher educators. In light of this, it is still important to examine the lifelong learning skills, competencies, and tendencies of all stakeholders in the educational setting. The previous studies analyzed different factors that may shape lifelong learning and/or lifelong learning abilities with all possible participants. Considering the geographical context, more research must be conducted on the three research trends in lifelong learning in Asia as opposed to Europe. This will strengthen the generalizability of findings to specific target groups such as students, teachers, and teacher trainers in the specific area.

Nevertheless, it must be emphasized that our study is not without limitations. Our review may have overlooked several empirical studies that were not in Scopus, WoS, or ProQuest because we selected only open-access articles indexed in these databases. Additional research may have a different effect on the results. Neither the details of the research instruments nor the findings of each study can be examined in detail.

Therefore, we recommend that subsequent systematic reviews and meta-analyses in lifelong learning incorporate articles indexed in other databases. Researchers may also conduct future reviews examining the history and psychometrics of research instruments used in lifelong learning and considers the results of each empirical study. However, a comparison of study findings in the Asian context continues to be a challenge because not enough research has been conducted in all possible lifelong learning research areas. Considering the impact of COVID-19, lifelong learning research in new learning communities, environments, or organizations may be conducted to capture updated information.

## Conclusion

This literature review aimed to identify concepts, theories, issues, trends, and research methodologies associated with lifelong learning in educational research. Our findings addressed concepts, lifelong learning policies, lifelong learning competencies, and formal, nonformal, and informal. The studies included in this review highlighted that a strong theory of lifelong learning has yet to be developed and applied. In addition, we deductively examined three common research trends: issues with basic concepts or guiding principles of lifelong learning, problems surrounding lifelong learning capacities, and challenges regarding variables that affect lifelong learning and/or lifelong learning capacities. Regarding methodology, we examined the techniques, tools, data analysis, and participants included in lifelong learning studies. Overall, educational researchers must continue to conduct more mixed methods studies, focusing on the Asian context.

## References

[CR1] Adams D (2007). Lifelong learning skills and attributes: The perceptions of Australian secondary school teachers. Issues in Educational Research.

[CR2] Bagnall RG (2017). A critique of Peter Jarvis’s conceptualisation of the lifelong learner in the contemporary cultural context. International Journal of Lifelong Education.

[CR3] Bath DM, Smith CD (2009). The relationship between epistemological beliefs and the propensity for lifelong learning. Studies in Continuing Education.

[CR4] Beighton C (2021). Biopolitics and lifelong learning: The vitalistic turn in English further education discourse. International Journal of Lifelong Education.

[CR5] Black S (2021). Lifelong learning as cruel optimism: Considering the discourses of lifelong learning and techno-solutionism in South African education. International Review of Education.

[CR6] Buza L, Buza H, Tabaku E (2010). Perceptıon of lifelong learning in higher education. Problems of Education in the 21st Century.

[CR7] Cefalo R, Kazepov Y (2018). Investing over the life course: The role of lifelong learning in a social investment strategy 1. Studies in the Education of Adults.

[CR8] Cort P (2009). The EC discourse on vocational training: How a “common vocational training policy” turned into a lifelong learning strategy. Vocations and Learning.

[CR9] Coşkuna YD, Demirel M (2010). Lifelong learning tendency scale: The study of validity and reliability. Procedia - Social and Behavioral Sciences.

[CR10] Council of the European Union. (2018). Council recommendation on key competences for lifelong learning. In *Official Journal of the European Union* 61(2). https://cutt.ly/MKKtVUN

[CR11] Crick RD, Broadfoot P, Claxton G (2004). Developing an effective lifelong learning inventory: The ELLI Project. Assessment in Education: Principles, Policy and Practice.

[CR12] Cronin C (2011). Doing your literature review: Traditional and systematic techniques. Evaluation & Research in Education.

[CR13] Deveci T (2019). Interpersonal communication predispositions for lifelong learning: The case of first year students. Journal of Education and Future-Egitim Ve Gelecek Dergisi.

[CR15] Deveci T (2022). UAE-based first-year university students’ perception of lifelong learning skills affected by COVID-19. Tuning Journal for Higher Education.

[CR16] Dewey, A., & Drahota, A. (2016). *Introduction to systematic reviews: online learning module*. Cochrane Training. Available at https://Training.Cochrane.Org/Interactivelearning/Module-1-Introduction-Conducting-Systematic-Reviews. Retrieved March 6, 2020.

[CR17] do Nascimento, D. V., Valdés-Cotera, R., & (Germany), U. I. for L. L. (UIL). (2018). Promoting Lifelong Learning for All: The Experiences of Ethiopia, Kenya, Namibia, Rwanda and the United Republic of Tanzania. UIL Publications Series on Lifelong Learning Policies and Strategies: No. 5. In *UNESCO Institute for Lifelong Learning* (Issue 5). https://search.ebscohost.com/login.aspx?direct=true&db=eric&AN=ED590194&lang=es&site=ehost-live

[CR18] Do T-T, Thi Tinh P, Tran-Thi H-G, Bui DM, Pham TO, Nguyen-Le V-A, Nguyen T-T (2021). Research on lifelong learning in Southeast Asia: A bibliometrics review between 1972 and 2019. Cogent Education.

[CR20] Edwards R, Usher R (1998). Lo(o)s(en)ing the boundaries: From “education” to “lifelong learning”. International Journal of Phytoremediation.

[CR21] Elken M (2015). Developing policy instruments for education in the EU: The European Qualifications Framework for lifelong learning. International Journal of Lifelong Education.

[CR22] Eschenbacher S, Fleming T (2020). Transformative dimensions of lifelong learning: Mezirow, Rorty and COVID-19. International Review of Education.

[CR23] European Commission. (2001). Making a European area of lifelong learning a reality—communication from the commission, COM(2001) 678 final. *Eric*. http://scholar.google.com/scholar?hl=en&btnG=Search&q=intitle:No+Title#0%5Cnhttp://eric.ed.gov/?id=ED476026

[CR25] Green A (2002). The many faces of lifelong learning: Recent education policy trends in Europe. Journal of Education Policy.

[CR26] Grokholskyi VL, Kaida NI, Albul SV, Ryzhkov EV, Trehub SY (2020). Cognitive and metacognitive aspects of the development of lifelong learning competencies in law students. International Journal of Cognitive Research in Science, Engineering and Education.

[CR28] Ivanova I (2009). A good adult educator as an important factor in the lifelong. Education And Training.

[CR29] James D (2020). Is lifelong learning still useful? Disappointments and prospects for rediscovery. Journal of Education and Work.

[CR30] Jarvis P (2009). The Routledge international handbook of lifelong learning.

[CR31] Karalis T (2009). Lifelong learning and preschool education: odd couple or eclectic relationship?. Problems of Education in the 21st Century.

[CR32] Karataş K, Şentürk C, Teke A (2021). The mediating role of self-directed learning readiness in the relationship between teaching-learning conceptions and lifelong learning tendencies. Australian Journal of Teacher Education.

[CR33] Kuit, T., & Fildes, K. (2014). Changing curriculum design to engage students to develop lifelong learning skills in biology. *International Journal of Innovation in Science and Mathematics Education*, *22*(2), 19–34. https://ro.uow.edu.au/cgi/viewcontent.cgi?article=3598&context=smhpapers

[CR34] Kwan, E., MacLeod, S., Chandler, M., & Fox, T. (2017). Report on a literature review of reforms related to the 2006 European Framework of Key Competences for lifelong learning and the role of the Framework in these reforms. In *European Commission, Bruselas. Recuperado de *https://bit.ly/2Ixsj5F.

[CR35] Lavrijsen J, Nicaise I (2017). Systemic obstacles to lifelong learning: The influence of the educational system design on learning attitudes. Studies in Continuing Education.

[CR36] Mallett R, Hagen-Zanker J, Slater R, Duvendack M (2012). The benefits and challenges of using systematic reviews in international development research. Journal of Development Effectiveness.

[CR37] Mandal S (2019). The rise of lifelong learning and fall of adult education in india. London Review of Education.

[CR38] Matsumoto-Royo K, Ramírez-Montoya MS, Glasserman-Morales LD (2022). Lifelong learning and metacognition in the assessment of pre-service teachers in practice-based teacher education. Frontiers in Education.

[CR39] Maxwell B (2014). Improving workplace learning of lifelong learning sector trainee teachers in the UK. Journal of Further and Higher Education.

[CR40] Moore T, Shaffer SC (2017). Awakening the learner within: purposeful prompts and lifelong learning measures in a first-year composition course. Journal of the Scholarship of Teaching and Learning.

[CR41] Muller R, Beiten S (2013). Changing learning environments at university? Comparing the learning strategies of non-traditional European students engaged in lifelong learning. Journal of Educational Sciences & Psychology.

[CR42] Nacaroglu O, Kizkapan O, Bozdag T (2021). Investigation of lifelong learning tendencies and self-regulatory learning perceptions of gifted students. Egitim ve Bilim.

[CR43] Nicoll K, Fejes A (2011). Lifelong learning: A pacification of “know how”. Studies in Philosophy and Education.

[CR44] Omirbayev S, Akhmed-Zaki D, Mukhatayev A, Biloshchytskyi A, Kassenov K, Faizullin A (2021). The conceptual foundations of lifelong learning in Kazakhstan: Process modeling. International Journal of Emerging Technologies in Learning.

[CR45] Oosterbeek H, Patrinos HA (2009). Financing lifelong learning. Empirical Research in Vocational Education and Training.

[CR46] Osborne M, Borkowska K (2017). A European lens upon adult and lifelong learning in Asia. Asia Pacific Education Review.

[CR47] Page MJ, McKenzie JE, Bossuyt PM, Boutron I, Hoffmann TC, Mulrow CD, Shamseer L, Tetzlaff JM, Moher D (2021). Updating guidance for reporting systematic reviews: Development of the PRISMA 2020 statement. Journal of Clinical Epidemiology.

[CR48] Panitsides, E. A. (2014). Lifelong Learning as a lever for tackling the ageing phenomenon in the European Union: new challenges, new tools. *Journal of Educational Sciences and Psychology*, *IV*(1). https://www.researchgate.net/publication/265330401_Lifelong_Learning_as_a_lever_for_tackling_the_ageing_phenomenon_in_the_European_Union_New_challenges_new_tools

[CR49] Rambla X, Kazepov Y, Jacovkis J, Alexander L, Amaral PD, M.  (2020). Regional lifelong learning policies and the social vulnerability of young adults in Girona and Vienna. International Journal of Lifelong Education.

[CR50] Sahin M, Akbasli S, Yelken TY (2010). Key competences for lifelong learning: The case of prospective teachers. Educational Research and Reviews.

[CR51] Sen N, Durak HY (2022). Examining the relationships between english teachers’ lifelong learning tendencies with professional competencies and technology integrating self-efficacy. Education and Information Technologies.

[CR52] Shin Y-S, Jun J (2019). The hierarchical effects of individual and organizational variables on elementary school teachers’ lifelong learning competence. International Electronic Journal of Elementary Education.

[CR53] Simmons R, Walker M (2013). A comparative study of awarding organisation and HEI initial teacher training programmes for the lifelong learning sector in England. Professional Development in Education.

[CR54] Steffens K (2015). Competences, learning theories and moocs: Recent developments in lifelong learning. European Journal of Education.

[CR55] Sunthonkanokpong W, Murphy E (2019). Quality, equity, inclusion and lifelong learning in pre-service teacher education. Journal of Teacher Education for Sustainability.

[CR56] Theodosopoulou M (2010). The challenge of developing strategic lifelong learning in the school community. Problems of Education in the 21st Century.

[CR57] Tsatsaroni A, Evans J (2014). Adult numeracy and the totally pedagogised society: PIAAC and other international surveys in the context of global educational policy on lifelong learning. Educational Studies in Mathematics.

[CR58] Tuijnman, A., Organisation for Economic Co-operation and Development., OECD Education Committee. Meeting (4th : 1996 : Paris, F. (1996). *Lifelong learning for all : Meeting of the Education Committee at Ministerial level, 16–17 January 1996.* (Vol. 9). http://hdl.voced.edu.au/10707/97779

[CR59] Tuparevska E, Santibáñez R, Solabarrieta J (2020). Equity and social exclusion measures in EU lifelong learning policies. International Journal of Lifelong Education.

[CR60] Tuparevska E, Santibáñez R, Solabarrieta J (2020). Social exclusion in EU lifelong learning policies: Prevalence and definitions. International Journal of Lifelong Education.

[CR61] Tyson LS, Vega VW (2019). Why we need to talk about lifelong learning and intercultural universities. London Review of Education.

[CR62] UIL, R. Y. (2017). Lifelong learning in transformation : Promising practices in Southeast Asia. In *UNESCO Institute for Lifelong Learning* (Issue 4). ERIC.

[CR63] Usher R (2015). Riding the lines of flight. European Journal for Research on the Education and Learning of Adults.

[CR64] Valiente O, Capsada-Munsech Q, de Otero JPG (2020). Educationalisation of youth unemployment through lifelong learning policies in Europe. European Educational Research Journal.

[CR65] Valiente O, Lowden K, Capsada-Munsech Q (2020). Lifelong learning policies for vulnerable young adults in post-recession Scotland. British Journal of Sociology of Education.

[CR66] Walters, S., Yang, J., & Roslander, P. (2014)* Lifelong Learning in Selected African Countries: Ethiopia, Kenya, Namibia, Rwanda and Tanzania. UIL Publication Series on Lifelong Learning Policies and …*. ERIC. https://eric.ed.gov/?id=ED560506%0Ahttps://files.eric.ed.gov/fulltext/ED560506.pdf

[CR67] Watson, L. (2003). *Lifelong learning in Australia*. https://www.semanticscholar.org/paper/Lifelong-learning-in-Australia-Watson/30be62bbe2448a4f9df723b70c2e2ab0f96cc854

[CR68] Yang, J., Schneller, C., Roche, S., & (Germany), U. I. for L. L. (UIL). (2015). The Role of Higher Education in Promoting Lifelong Learning. UIL Publication Series on Lifelong Learning Policies and Strategies: No. 3. In *UNESCO Institute for Lifelong Learning*. ERIC. https://login.proxy.hil.unb.ca/login?url=https://search.ebscohost.com/login.aspx?direct=true&db=eric&AN=ED564050&site=ehost-live&scope=site

[CR69] Yen CJ, Tu CH, Sujo-Montes LE, Harati H, Rodas CR (2019). Using personal learning environment (PLE) management to support digital lifelong learning. International Journal of Online Pedagogy and Course Design.

[CR70] Yorozu, R. (2017). Lifelong learning in transformation: Promising practices in Southeast Asia: Brunei Darussalam, Cambodia, Indonesia, Lao People’s Democratic Republic, Malaysia, Myanmar, Philippines, Singapore, Thailand, Timor-Leste and Viet Nam. In *UNESCO Insitute for Lifelong Learning* (Issue 4). UNESCO Insitute for Lifelong Learning. https://unesdoc.unesco.org/ark:/48223/pf0000253603

[CR71] Zuhairi A, Hsueh ACT, Chiang I-CN (2020). Empowering lifelong learning through open universities in Taiwan and Indonesia. Asian Association of Open Universities Journal.

